# Construction of a Chitosan/ZnO-Based Light-Resistant Coating System to Protect Dyed Wood from Ultraviolet Irradiation via Layer-by-Layer Self-Assembly

**DOI:** 10.3390/ijms232415735

**Published:** 2022-12-12

**Authors:** Zhe Luo, Yang Zhang

**Affiliations:** 1 National Forestry and Grassland Engineering Technology Center for Wood Resources Recycling, Beijing Forestry University, Beijing 100083, China; 2 Beijing Key Laboratory of Wood Science and Engineering, Beijing Forestry University, Beijing 100083, China

**Keywords:** dyed wood, UV, chitosan, ZnO, photochromic, assembly

## Abstract

Wood dyeing is an effective way to alleviate the supply-demand imbalance of valuable wood and improve the surface decoration of fast-growing wood. However, applications of dyed wood are limited due to the susceptibility of dyes and wood to photo-discolor and degrade under light irradiation. Thus, the improved weather resistance of dyed wood is crucial. To prevent photochromic discoloration of dyed wood, an anti-photochromic coating structure was constructed via layer-by-layer self-assembly (LbL) using chitosan and zinc oxide (ZnO). The results showed that the surface color difference of treated dyed wood was reduced by approximately 84.6% after the first 2 h of irradiation under the following conditions °C: temperature (50 °C), relative humidity (55%), and irradiation intensity (550 W/m^2^). However, the color of untreated dyed wood drastically changed at this stage. The reason for the decrease was that the redness and yellowness of treated dye wood were significantly reduced. The deposition of ZnO onto treated dyed wood helped to protect the wood from UV light irradiation. Chitosan bridged the dyes and complexed ZnO to enhance UV resistance. This study provides valuable information for the protection of dyed wood against light discoloration that can be used as an interior and exterior decorative material.

## 1. Introduction

The 2020 Global Forest Resources Assessment issued by the Food and Agriculture Organization of the United Nations showed that the world’s forest growing stock was declining. According to this assessment, the world lost 178 million ha of forests between 1990–2020 [1]. Approximately 30% of the world’s total forest land area is used for the production of wood and non-wood products [1,2]. Precious woods are widely produced for their rustic colors, textures, and comfort. However, the commodity trade of precious timber is constrained by the carrying capacity of forest ecosystems [3]. Precious woods have a long-term growth cycle, which increases the cost of production and ultimately diminishes resources. Fast-growing wood, such as poplar, has a short-term growth cycle and is widely used; however, its poor color appearance makes it difficult to meet consumer standards and demand [4]. As a result, the dyeing of fast-growing wood to imitate high-performance precious timber can improve the utilization and commercial quality of fast-growing wood. Wood is dyed by uniformly penetrating dyes and other additives through the surface and interior of the wood. Due to the existence of unsaturated groups in dyes (e.g., carbonyl groups, phenolic hydroxyl groups, and benzene ring structures) [5], dyed wood is more susceptible to photo-oxidation or photo-reduction (degradation reactions by ultraviolet radiation) [6,7,8]. Thus, dyed wood is more prone to photo-discoloration than undyed wood [9]. Improving the light resistance of dyed wood is critical for extending its service life and protecting the growth of precious wood. Furthermore, it has significant implications for promoting the rapid development of plantation forests, conserving natural resources, and ensuring sustainable development of the wood processing sector.

In general, the ultraviolet (UV) resistance of wood can be improved by adding inorganic or organic sunscreens to the wood surface [10]. Organic UV absorbers and hindered amine light stabilizers are conventional methods used to protect wood. Priya Bisht et al. prepared transparent wood composites with UV absorbers (2-(2H-Benzotriazol-2-yl)-4,6-di-tert-pentylphenol). The findings showed that the addition of UV absorbers greatly reduced the discoloration and photodegradation of wood composites [11]. Neng Li et al. showed that outdoor bamboo coatings with 5% organic UV absorbers could effectively reduce the photodegradation of substrates and coatings [12]. Saeid Nikafshar et al. found that the use of organic UV absorbers minimized undesirable effects of UV light on the mechanical strength of epoxy-amine systems when exposed to UV radiation [13]. However, organic UV absorbers can gradually decompose or transfer to the environment during the application [14,15]. Thus, organic UV absorbers cannot be used as long-term wood protectors. In addition, organic UV absorbers can pose potential threats to the ecosystem owing to their toxicity [16]. The short absorption band of organic UV absorbers for UV light can also affect the transparency of the coating. Zinc oxide (ZnO), a common inorganic UV absorber, has better UV absorption in both the ultraviolet radiation A (UVA) and ultraviolet radiation B (UVB) wavelengths. Because of the increased specific surface area and smaller particle size of ZnO nanoparticles [17], they exhibit enhanced UV absorption and good reflective effects of the coating. ZnO exhibits better long-term sustainability of the UV absorption effect than organic UV absorbers.

Sunscreens with ZnO as the main ingredient are widely commercialized. Similarly, many researchers have focused on the utilization of nanomaterials, such as ZnO, for the protection of wood surfaces. For instance, Sreeja Nair et al. prepared coatings by dispersing nano-metal oxides, which, in the presence of nanoparticles, enhanced the resistance of wood to UV degradation and prevented photo-yellowing [18]. Lizhuo Kong et al. grew rod-shaped ZnO nanoparticles in wood by in situ assembly, and the grown nanoparticles enhanced the light stability, flame retardancy, and water resistance of the wood [19]. However, the fabrication of inorganic UV-resistant coatings on dyed wood surfaces is less reported. More intense light absorption capacity of dyed wood can be attributed to the presence of structures that are more unsaturated [20]. The chemical structures of dyes and wood formed a combined complex able to absorb sunlight [9]. The deposition of ZnO on the surface of dyed wood is difficult due to the various organic and inorganic substances in ZnO and dyes. Chitosan is a natural polysaccharide that is frequently used as a high-efficient adsorbent for dyes or as dyeing support during the wood dyeing process [21,22,23]. The dyes are fixed by ionically bonding the amino groups in the chitosan molecules to the sulfonic acid groups in the dyes [22,24]. In addition, chitosan is a metal complexing agent, which can be coordinated with metals to form complexes [25]. The addition of cellulose nanocrystal/ZnO as a filler for poly(vinyl alcohol)/chitosan films by Susan Azizi et al. significantly improved the mechanical strength and UV absorption of poly(vinyl alcohol)/chitosan films using matrix-filler interactions [26]. Wei Ma et al. and Yeon Ho Kim et al. prepared biocidal materials composed of ZnO and chitosan and cellulose, respectively. The prepared materials exhibited excellent antibacterial effects [27] and UV stability [28]. However, ZnO was easily agglomerated, and when it was added to the chitosan solution to prepare a film, the dispersion was poor, leading to a lower UV absorption effect [29]. As a result, the anionic ZnO dispersion was configured to overcome the ZnO agglomeration problem. Meanwhile, the cationic and film-forming properties of chitosan solution were used to construct an anti-photochromic coating with positive and negative charge adsorption on the surface of dyed wood. Accordingly, the focus of this work was on a facile approach to solve the problems of inorganic-organic interfacial compatibility and ZnO dispersion, i.e., layer-by-layer (LbL) self-assembly to prepare a bio-based anti-photochromic coating. The coating both fixed the dyes with chitosan and absorbed UVA and UVB wavelengths with ZnO. The coating showed excellent UV resistance and substantially reduced the differences in color produced via photochromic discoloration of dyed wood.

In this study, given the favorable properties of chitosan, it was used to bridge dyes and metal oxide for the construction of a light-resistant coating system for dyed wood. The coatings were constructed using LbL self-assembly. Different types of chitosan derivatives were selected for the cationic coating, and anionic dispersants were used to prepare a homogeneous and stable anionic coating of ZnO. The effects of different chitosan derivatives and ZnO concentrations on the light resistance of dyed wood were investigated. Additionally, the structure and protective mechanism of the light-resistant coatings on dyed wood were evaluated.

## 2. Results and Discussion

### 2.1. Optimal LbL Process of Treated Wood

**Figure 1:**
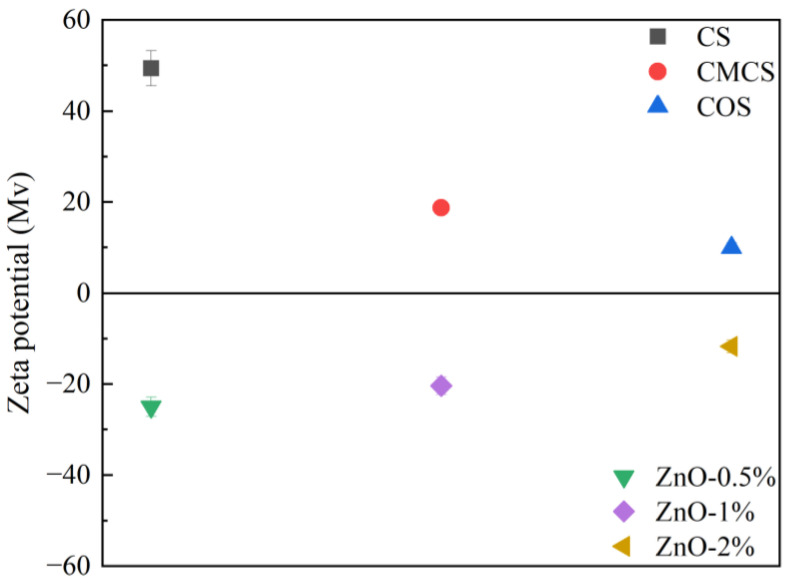
Zeta potentials of chitosan (CS), carboxymethyl chitosan (CMCS), and chitosan oligosaccharide (COS), with ZnO (0.5%, 1%, and 2%).

**Figure 2:**
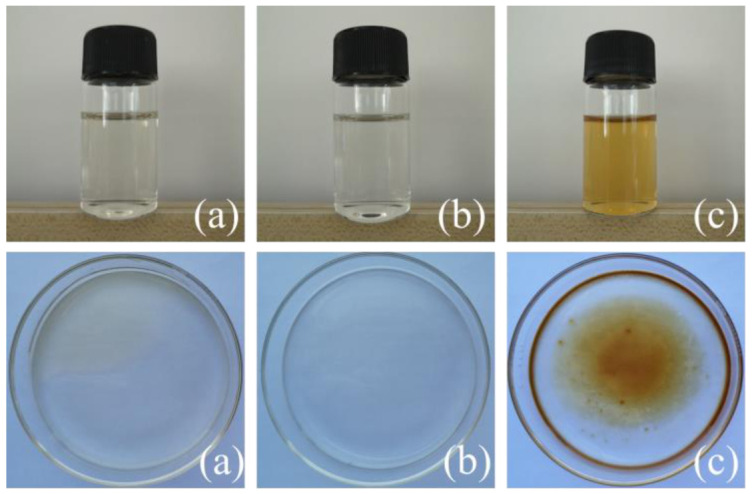
Photographs of the solutions (**top**) and films (**bottom**) prepared with 1% (**a**) chitosan, (**b**) carboxymethyl chitosan, and (**c**) chitooligosaccharide from left to right.

The anti-UV effects of treated wood were correlated with the cationic layer and concentration of ZnO in the anionic layer of the LbL. Different forms of chitosan formed active groups (such as amino groups) and different aggregations of molecular chains [30,31] and slightly changed the appearance of dyed wood when coated. The luminosity (L), red-green index (a), and yellow-blue index (b) together form the color of dyed wood, where the luminosity represents the lightness and darkness of the specimen, and the red-green and yellow-blue indexes indicate the bias toward red and green or yellow and blue, respectively. The different changes of the L, a, and b values before and after irradiation result in the color difference (∆E), thus ∆E represents the total color change of specimens. The changes in ∆E over time for untreated dyed wood and treated wood compounded with different chitosan forms are shown in Table 1. The color difference of treated wood was significantly diminished compared to untreated dyed wood, especially in the accelerated pre-irradiation period (0–2 h of irradiation). The pre-irradiation color difference of wood treated with carboxymethyl chitosan compound was only 0.81, indicating that it performed best among the three compounds. The color difference values of the three sample treatments were close at different irradiation times (5–50 h), and all samples displayed excellent UV resistance (Table 1). The zeta potentials of the three forms of chitosan exhibited cationic properties (Figure 1). This phenomenon was because the amino groups on the molecular chains of chitosan were protonated under acidic conditions and then became positively charged. Meanwhile, chitosan and chitosan-oligosaccharide showed light yellow when the colors of the three groups of coatings were compared at 1%. Only carboxymethyl chitosan was colorless and transparent (Figure 2), which showed lesser effects of coloration of the dyed wood.

**Figure 3:**
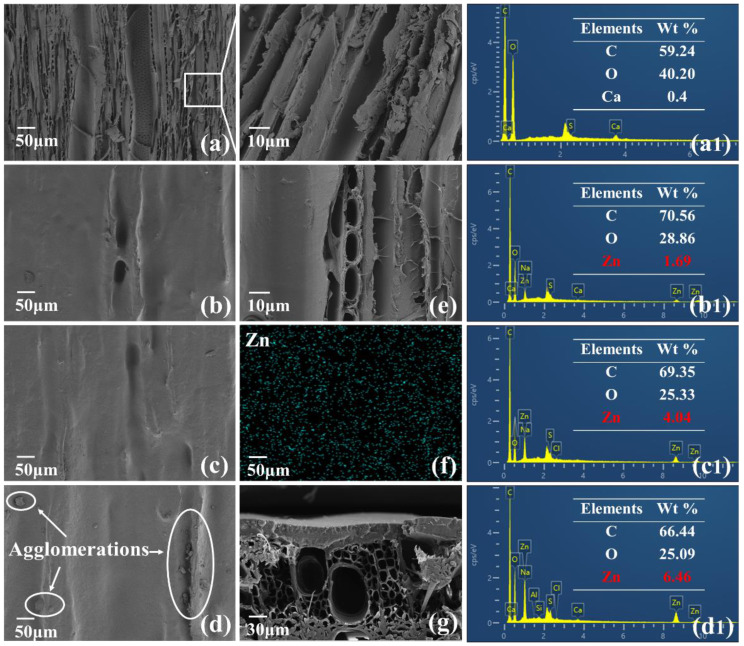
SEM micrographs of (**a**) dyed wood and (**b**) dyed wood treated with ZnO-0.5%, (**c**) treated with ZnO-1%, (**d**) treated with ZnO-2%; (**e**) dyed wood coated with carboxymethyl chitosan; (**f**) EDS mapping of (**c**) Zn; (**g**) cross-section of treated wood; the EDS spectra of (**a1**) image (**a**), (**b1**) image (**b**), (**c1**) image (**c**) and (**d1**) image (**d**).

The concentration of ZnO in anionic dispersion determined the distribution of ZnO on the surface of dyed wood and the actual loading. In addition, the concentration of ZnO affected the UV absorption effect. The morphologies of the coatings are shown in Figure 3. The microstructure of dyed wood was covered after the LbL process (Figure 3a,b). Different dispersions of ZnO were observed on the surface of the dyed wood (Figure 3b–d). As shown in Figure 3d, 0.5% and 1% ZnO were uniformly dispersed, whereas 2% ZnO showed some agglomeration. The elemental content distribution spectra (Figure 3a1–d1) showed that the ZnO loading on the dyed wood surfaces increased with increasing ZnO concentration. Moreover, the zeta potential gradually increased with increasing ZnO concentration (Figure 1). This phenomenon may be attributed to the positively charged surface of ZnO nanoparticles at pH < 9.5 [32]. The negative charge of the polyacrylamide molecules adsorbed on the surface of ZnO was partially neutralized due to increasing ZnO in the solution [33].

**Figure 4:**
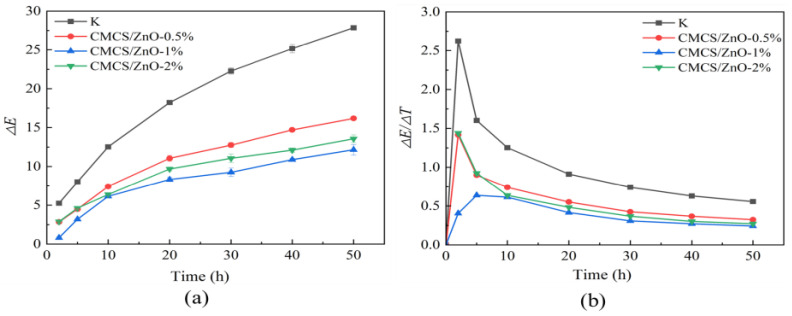
∆E changes (**a**) and color variation rates (**b**) of untreated dyed wood (K) and treated wood (CMCS/ZnO-0.5%, CMCS/ZnO-1%, and CMCS/ZnO-2%) under 200–800 nm UV light irradiation.

Figure 4 shows the color variations of the dyed wood treated with three groups. CMCS/ZnO-1% color slightly changed after the first 2 h of irradiation. At 2 h and 5 h of irradiation, the ∆E values of CMCS/ZnO-0.5% and CMCS/ZnO-2% were similar. However, the ∆E value of CMCS/ZnO-2% was higher than that of CMCS/ZnO-0.5% after 10 h irradiation. Moreover, the ∆E values of CMCS/ZnO-2% were higher than those of CMCS/ZnO-1% (Figure 4a). This phenomenon could be attributed to the excessive ZnO agglomeration on the treated wood surface and the generation of more photocatalytic activity, which in turn reduced the UV protection effect [34]. Therefore, the optimal color difference values of treated wood were integrated (Figure 4), and carboxymethyl chitosan and 1% ZnO dispersion were selected as the cationic and anionic coatings of treated wood, respectively, for subsequent experiments.

The coating consisting of carboxymethyl chitosan and ZnO was self-assembled on the surface of dyed wood, and the film structure on dyed wood was observed using SEM. Figure 3a,c,e show images of unmodified dyed wood, dyed wood coated with carboxymethyl chitosan, and dyed wood coated with carboxymethyl chitosan compounded with ZnO, respectively. The surface of dyed wood after carboxymethyl chitosan deposition (Figure 3e) became significantly smoother than that of unmodified dyed wood due to the adhesion of dye molecules. This phenomenon can be attributed to the binding of chitosan to the dyes [22] and the film-forming property of chitosan, indicating that chitosan was effectively deposited on the dyed wood surface. ZnO was uniformly dispersed on the surface of the dyed wood (Figure 3f), and a smooth coating covered the entire surface (Figure 3c). A tightly connected film of average 31.97 ± 1.35 µm was formed on the dyed wood surface after the deposition of the cationic and anionic solutions (Figure 3g), respectively, which might be attributed to the electrostatic gravitational force of LbL.

### 2.2. Evaluation of the Anti-Ultraviolet Effect of LbL Coatings on Dyed Wood

**Figure 5:**
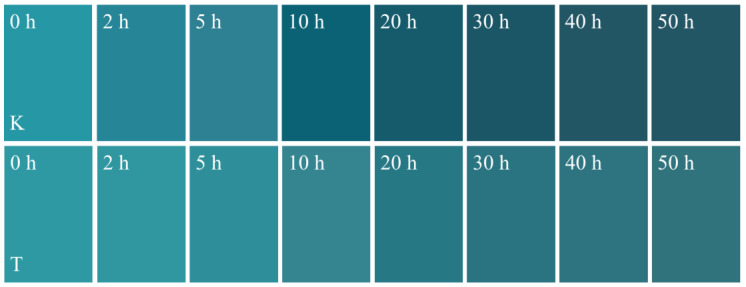
Visual assessment of the untreated dyed wood (K) and treated wood (T) after 50 h UV irradiation.

UV resistance is one of the most important indicators when evaluating the weatherability of dyed wood [35]. The UV resistance of dyed wood was characterized using variations in its surface color after UV radiation. Table 2 compares the UV protection effect of this work with other similar studies. The photo discoloration of the dyed wood was more intense due to the combined absorption of light by the wood and dyes. However, the ∆E reduction of treated dyed wood reached 56.4% after irradiation, which was better than or equivalent to the UV protection reported in similar studies. As shown in Figure 5, the surface brightness of the dyed wood decreased significantly with increasing irradiation times, and the most significant color change was observed in the first 10 h of irradiation. In contrast, a slight change was observed in the surface color of the dyed wood treated with carboxymethyl chitosan and zinc oxide. At 20–50 h, the color changes on the surfaces of untreated and treated dyed woods were not dramatic as in the first 10 h irradiation. As shown in Figure 4a, the ∆E of untreated and treated dyed woods always tended to increase. However, different patterns of variation were observed in the untreated and treated dyed woods. The color difference in treated wood initially increased and then stabilized, while that of the untreated dyed wood continued to increase. Meanwhile, the color difference in the untreated dyed wood was consistently more than that of treated wood for 50 h irradiation. The rate of color change in both groups first increased and then decreased, with a maximum value observed after 2 h of irradiation time. Subsequently, the rate of color change gradually decreased.

**Figure 6:**
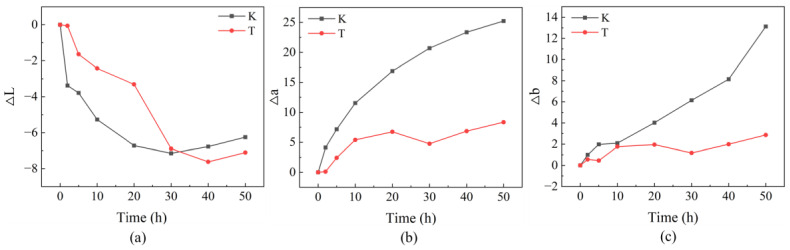
(**a**) ∆L, (**b**) ∆a, and (**c**) ∆b changes of dyed wood (K) and treated wood (T) with respect to UV light irradiation.

Figure 6a illustrates changes in the values of lightness (ΔL) for untreated and treated dyed wood. Both untreated and treated wood showed negative ∆L values after 50 h of exposure, –6.2 and –7.1, respectively. The sharp change in brightness of untreated wood occurred in the first two hours of irradiation, while that of treated wood was 20–30 h. Both displayed stable ∆L values after 30 h of irradiation. However, the pre-darkening of treated wood was much less than untreated wood. Changes in Δa and Δb for untreated and treated wood are shown in Figure 6b,c. The untreated wood showed severe darkening indicated by negative ∆L values, which, together, increased ∆a and Δb. The ∆a and ∆b values of untreated wood after 50 h irradiation were 25.2 and 13.1, respectively. However, the ∆a and ∆b values for treated wood were only 8.3 and 2.9.

Rapid changes in the color of untreated dyed wood observed in the first 2 h of irradiation were defined as pre-irradiation, which agrees with previous descriptions [9]. Initially, the dyes, lignin, and some extractives in untreated dyed wood absorbed light energy, which resulted in oxidation and degradation reactions, thereby changing the color of dyed wood [9,41]. The brightness of untreated and treated dyed wood was significantly reduced (Figure 6a), with a gradually darkened surface. The increase in the red-green index of untreated dyed wood was also higher than that of treated wood (Figure 6b), presenting as a red-turning trend. More moderate yellowing trends were observed in the untreated and treated dyed woods relative to the yellow-blue index early in the irradiation scheme (Figure 6c).

At 5–50 h of irradiation, i.e., in the middle and final stages of irradiation, the color change rates of the untreated and treated dyed woods (Figure 4b) displayed decreased trends and gradually leveled off. However, the color difference in the untreated dyed wood always increased compared to treated dyed wood (Figure 4a). Compared with treated dyed wood, untreated dyed wood had more pronounced reddening and yellowing despite a similar change in brightness (Figure 6). Thus, the overall color variation remained significantly high. In contrast, the brightness, red-green index, and yellow-blue index of treated dyed wood showed a slowly decreasing or increasing trend toward flattening in the middle and final stages of irradiation. In general, the color variation of untreated dyed wood during irradiation can be attributed to a remarkable reduction in brightness and then yellowing, which is consistent with the observed color changes. However, the change in color in the treated dye wood was gradually stabilized, indicating that the treated dye wood was only slightly discolored, even after prolonged radiation exposure. The results show that superior UV resistance of treated dyed wood was achieved by reducing the reddening and yellowing in the middle and final stages of light irradiation.

### 2.3. Effect of LbL Coating on the Surface Reflectance of Dyed Wood

**Figure 7:**
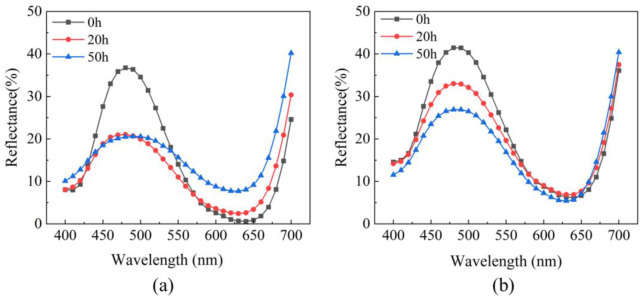
(**a**) Reflectance spectra of untreated dyed wood and (**b**) treated dyed wood irradiated at 0, 20, and 50 h.

The surface color saturation of dyed wood is closely correlated with its reflectance properties. As shown in Figure 7, fluctuating spectral reflectance curves with increasing wavelength were observed in untreated and treated dyed wood, presented in a pattern of variation in the sine function. The spectral reflectance curves of both groups of specimens were shifted downward overall at the end of 50 h irradiation, reducing the color saturation. Thus, the surface darkened, and L significantly declined. According to the relationship between color and wavelength [42], the reflectance was high in the range of 450–525 nm for the two groups of samples, corresponding to the blue color. By comparing the spectral reflectance curves of untreated and treated dyed woods, the reflectance of untreated dyed wood rapidly decreased in the range of 450–525 nm during the first 20 h irradiation and slightly changed after 20 h until the end of irradiation. However, the reflectance of the treated dye wood gradually decreased after 20 h. The proportion of yellow and red in the reflectance spectra increased in accordance with the theory of color complementarity and the color wavelength connection [43], thus decreasing their complementary light blue and purple in the range of 450–500 nm. This result also shows that the untreated dyed wood tended to be red and yellowing, especially in the middle and final stages of irradiation, consistent with the changes in ∆a and ∆b above. However, the treated dyed wood performed quite well in the range of 450–500 nm with almost no change.

**Figure 8:**
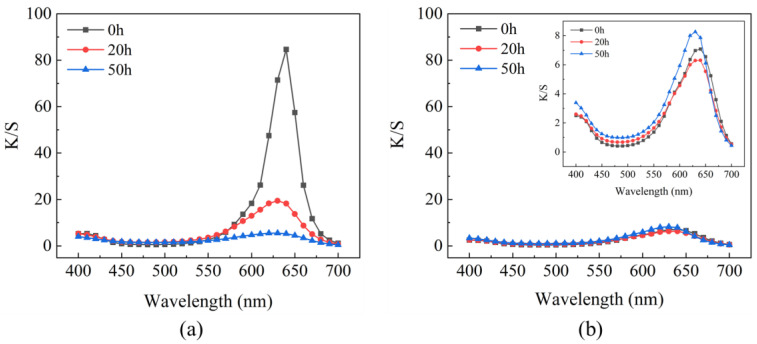
(**a**) K/S spectra of untreated dyed wood and (**b**) treated wood irradiated at 0, 20, and 50 h UV light.

The K/S curve of untreated dyed wood (Figure 8a) revealed that the K/S value of untreated dyed wood significantly decreased in the first 20 h of irradiation, indicating that the discoloration and degradation mainly occurred at this stage, consistent with previous literature [9]. The absorption peaks in the wavelength range of 600–650 nm widened and gradually flattened out, meaning that the structure of color-forming groups of untreated dyed wood changed and the absorption coefficient decreased, resulting in a hypochromic effect. The fluctuations in the K/S curve of treated dyed wood were minimal compared to untreated dyed wood (Figure 8b). The K/S curve of treated wood slightly shifted upward in the range of 600–650 nm at the end of the irradiation cycle. To some extent, the K/S values represented the color intensity of untreated dyed wood, i.e., the concentration of dye on the surface. By comparing the overall change in K/S values of untreated and treated dyed woods, a significant decrease in the color intensity of untreated dyed wood was found, indicating that some dye degradation had occurred. In contrast, treated dyed wood remained almost unchanged. Consequently, the treated dyed wood exhibited high UV protection by reducing the degradation of dyes.

### 2.4. FTIR Analysis of Untreated and Treated Dyed Woods

**Figure 9:**
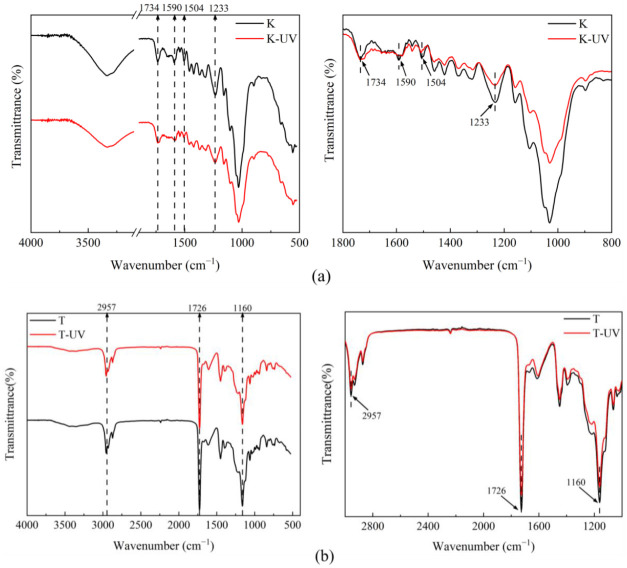
FTIR spectra of dyed wood (**a**) and treated wood (**b**) before and after UV irradiation.

In this study, changes in chemical composition on the surface of samples were examined using FTIR during the aging experiments. Figure 9a shows the infrared spectra obtained before and after 50 h full-band UV irradiation of untreated dyed woods. The band was assigned using the reference data cited in Table 3 [9,44,45]. The intensity of the C=C peaks at 1504 cm^−1^ and 1590 cm^−1^ was reduced in untreated wood, which reflected the degradation of lignin [46]. The lignin degradation was accompanied by the generation of more carbonyl structures and structural changes in the color-generating groups of dyed wood, resulting in considerable changes in surface color [46,47]. In addition, the reduction in the characteristic peak at 1233 cm^−1^ for untreated wood was attributed to the methoxy reaction on the benzene ring [48]. However, no changes in the characteristic peaks of lignin were observed on the surface of treated dyed wood, which could be attributed to a uniform and adequate coverage of untreated dyed wood by the coatings and thicker film layers. The coating reflected and absorbed UV light because of its thickness and the uniform distribution of ZnO particles on the surface, which improved the UV resistance of treated wood.

The UV shielding effect of the treated wood is mainly based on the absorption of UV light protected by ZnO [49,50]. The FTIR spectroscopy results of treated wood are shown in Figure 9b. The photostability of the treated dyed wood surface was influenced by the concentration of ZnO dispersion. When the coating was applied to dyed wood, the intensity of the C=O peak at 1726 cm^−1^ decreased, indicating that the coating slowed the generation of carbonyl-dominated unsaturated structures. The material surface is provided with more protection, thus the surface color is more stable [51]. In addition, extremely high concentrations of nanoparticles also led to the changes in photostability of the coating surface, as witnessed by the changes in peaks at 2957 cm^−1^ and 1160 cm^−1^, corresponding to the stretching vibrations of C-H and C-O, respectively. However, the intensities of the 2957 cm^−1^ and 1160 cm^−1^ peaks decreased, indicating that the coating underwent photo-oxidation under the photocatalytic effect of ZnO [37]. This procedure reduced the C-H and C-O groups and produced oxidation products, including hydrogen peroxide and carboxylic acid C=O [18,38]. In summary, the deposition of ZnO onto the surface of treated dyed wood through LbL was able to protect the wood from UV light irradiation. However, the addition of a light stabilizer is still required to prevent photocatalytic degradation of ZnO in the process.

## 3. Materials and Methods

### 3.1. Materials

Poplar veneers were purchased from Shandong, China. Wood specimens of typically 100 mm, 100 mm, and 1 mm in the longitudinal, radial, and tangential directions, respectively, were prepared from non-defective wood. Chitosan (80–95% DDA, CAS 9012-76-4), carboxymethyl chitosan (degree of substitution ≥ 90%, CAS 83512-85-0), chitosan oligosaccharides (CAS 148411-57-8), ZnO nanoparticles (<50 nm average particle size, CAS 1314-13-2), acetic acid (100%, CAS 64-19-7), polyacrylic amide (≥99%, CAS 9003-05-8), and acrylic acid (CAS 79-10-7) were purchased from Aladdin. Acid turquoise blue A (≥99.5%) was obtained from the second Plant Dye Chemical Company, Tianjin, China.

### 3.2. Preparation of Dyed Wood

Dyed wood was prepared following the procedure reported by Liu et al. [9]. Sulfuric acid (10 wt%) buffer was prepared by dissolving sulfuric acid in deionized water. The dyeing solution (0.15 wt%) was produced by adding acid turquoise blue A to deionized water, and the pH of the solution was adjusted with sulfuric acid buffer. Then, the cut veneers were impregnated with the dyeing solution under atmospheric pressure. The solution-impregnated veneers were distanced from each other to ensure the even application of dyes to the different veneers. The parameters of the dyeing process were set to a bath ratio of 1:20 at 90 °C for 2 h.

### 3.3. Preparation of Coating Solutions and LbL Assembled Coatings

**Figure 10:**
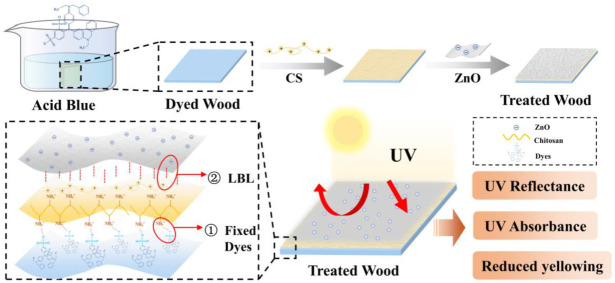
Preparation details and mechanism of the treated wood.

Chitosan solution was selected as the cationic coating of the LbL system. The 1wt% chitosan was dissolved in deionized water by adding acetic acid dropwise, stirred for 1 h until the chitosan was fully dissolved, and then sonicated for 30 min to form a clear solution. The nano-dispersion of ZnO was prepared by mixing anionic polyacrylamide and acrylic acid. After 1 h of continuous stirring at room temperature, the solution was sheared at 10 Krpm for 10 min in a high-shear emulsification machine. The resulting solution was used as the anionic coating of the LbL system. The dyed wood after LbL self-assembly was treated wood. Figure 10 shows the preparation process and mechanism of treated dyed woods.

The coating was applied onto the surface of dyed wood by successive depositions of chitosan and ZnO nanoparticles. To ensure a proper combination of wood and dyes, chitosan was selected as the first coating layer. The surface of the dyed wood was coated with 1 g of chitosan solution and allowed to dry for 20 min at room temperature. Afterward, the surface was coated with 1 g of ZnO dispersion. To study the influence of different factors on coatings, various chitosan derivatives and 0.5, 1, and 2 wt% of ZnO solution were used for the LbL system (Table 4).

### 3.4. Zeta Potential Measurements

Zeta potential tests were conducted using a zeta potential measuring machine (Nano Z). Potential tests were performed on chitosan, carboxymethyl chitosan, chitosan oligosaccharide solution, and ZnO dispersions (0.5%, 1%, and 2%). Each sample was measured at least five times, and the average results were recorded.

### 3.5. UV Resistance Test

Artificial accelerated aging was performed in a weathering chamber equipped with a xenon lamp (#SN-500, Beijing, China). The uncoated and coated dyed woods were exposed to the device to test surface light resistance. The wood samples were irradiated at 280–1100 nm for a total duration of 50 h. The experimental conditions were set: a blackboard temperature of 50 °C, relative humidity of 55%, and an irradiation intensity of 550 W/m^2^. The color difference of the samples was measured after irradiation times of 0, 2, 5, 10, 20, 30, 40, and 50 h.

### 3.6. Color Measurement

The surface chromaticity parameters were measured with a DF110 spectrophotometer. The International Commission on Illumination (CIE) LAB color system was set in a spectrophotometer (10° standard observer, D65 standard illuminant). Three parallel specimens were set for each experimental factor. Six different test locations were evenly selected for each sample, and their average values were recorded. The color parameters of samples were recorded before irradiation and after certain irradiation times. The total color difference (∆E) was used to evaluate the surface color of the dyed wood. ∆E was calculated using Equation (1):

(1) $$∆E={\left(∆L^{2}+∆a^{2}+∆b^{2}\right)}^{1/2}$$

where ∆E is the color difference of wood specimens before and after irradiation, L represents the lightness of wood specimens (expressed from 0–100 on the degree from black to white), a represents the red-green axis color quality index (where +a and −a indicate a color bias towards red and green, respectively), and b represents the yellow-blue axis color index (+b and −b indicate a color bias towards yellow and blue, respectively). ∆L, ∆a, and ∆b are the differences between the wood specimens before and after irradiation.

The degree of color change of samples after irradiation for a specific time is expressed by the color change rate (∆E/∆T). ∆T represented the time interval between measurements.

The light reflectance properties of dyed wood are expressed as reflectance spectra. The wavelength range of measurement was from 200–800 nm. As described by Liu et al. [9], the K/S curve was formed by converting the relationship between the absorption coefficient (K), scattering coefficient (S), and the reflectance (R) in the Kubelka–Munk equation model, following Equation (2):

(2) $$K/S=(1\;-{\;R)}^{2}/2R$$

where R is the reflectance, and K and S are the absorption coefficient and scattering coefficient, respectively.

### 3.7. Fourier Transform Infrared (FTIR) Analysis

The coated and uncoated dyed woods were analyzed using FTIR (Vertex 70, Bruker, Karlsruhe, Germany). Measurements were performed using 32 scans over a spectral range from 4000 to 500 cm^−1^.

### 3.8. Morphological Analysis

Scanning electron microscopy (SEM, JSM 6500F, Japan Electron Optics Laboratory, Tokyo, Japan) analysis was used to examine the surface topographies of samples. The distribution of particles and elements was investigated with an energy-dispersive spectrometer (EDS).

## 4. Conclusions

In this study, a photochromic-resistant coating was constructed on the surface of dyed wood through layer-by-layer self-assembly using chitosan and ZnO. The organic-inorganic coating was achieved through the electrostatic adsorption and complexation of chitosan with ZnO and the ionic bonding of chitosan with dyes, which effectively enhanced the UV protection performance of dyed wood. The coating, which was prepared by a facile and green method with remarkable photoprotective effects, has potential applications in the decorative material field.

Our study showed that carboxymethyl chitosan had more transparency compared to chitosan and chitosan oligosaccharides. The composite coating with ZnO-1% showed superior anti-UV effects while the composite coating with ZnO-2% showed reduced anti-UV effects and was prone to agglomeration. Therefore, the optimal process of carboxymethyl chitosan and ZnO-1% was chosen for the experiment. The color difference of the treated woods was consistently and significantly less than that of untreated dyed wood throughout the accelerated aging testing. In particular, the dyed wood treated with carboxymethyl chitosan and 1% ZnO exhibited excellent UV resistance in the pre-irradiation stage, with an approximately 84.6% reduction in color difference. However, the untreated dyed wood underwent drastic discoloration at this stage. From the middle to final stages of light irradiation, the color difference of treated dyed wood remained essentially stable while that of untreated dyed wood continued to increase. After irradiation, the color difference value of untreated dyed wood decreased from 27 to 12.

The morphology and chemical composition of the surface of untreated and treated dyed woods were characterized. The dramatic changes in the color of dyed wood were caused by a decrease in brightness as well as reddening and yellowing during irradiation. The reflectance and K/S curves showed that the LbL self-assembled coating effectively prevented the yellowing and reddening of dyed wood in the range of 450–500 nm while maintaining the color intensity of dyed wood in the range of 600–650 nm. This was attributed to the UV light absorption and reflection effects of coating, which effectively reduced the degradation of C=C groups and the formation of unsaturated structures by the coating. However, the presence of the photocatalytic activity of ZnO nanoparticles led to partial oxidation of the coating surface. Thus, the addition of a photostabilizer to the coating material is necessary to prevent the photocatalytic degradation effect of ZnO.

In this work, chitosan and ZnO were self-assembled into an environmentally friendly bio-based coating that exhibited excellent UV resistance on dyed wood. The self-assembly of colloidal particles allowed precise control of the optical properties and wettability of wood coating [52,53,54], different crystalline or non-crystalline arrangements [55,56,57,58], and modulation of the morphology of colloidal particles and assemblies [54,59,60,61,62]. However, colloidal particles are mostly synthetic polymers, limiting their application in the biological and medical fields. In contrast, chitosan is a degradable, biocompatible natural polymer that can also be used to immobilize metal nanomaterials. ZnO has some antibacterial properties in addition to its excellent optical properties. Therefore, the self-assembly of chitosan and ZnO is expected to be combined with the self-assembly of colloidal particles for the construction of bio-based responsive nanostructured surfaces and the preparation of antibacterial, bionanostic, and optical materials.

## Figures and Tables

**Table 1:** ∆E changes in different chitosan forms of composite-treated wood at different irradiation times.

Sample	2 h	5 h	10 h	20 h	30 h	40 h	50 h
K ^b^	5.25 ± 0.32 ^a^	8.01 ± 0.29	12.52 ± 0.29	18.22 ± 0.49	22.27 ± 0.64	25.16 ± 0.73	27.85 ± 0.55
CS/ZnO ^c^	1.42 ± 0.13	3.58 ± 0.18	7.54 ± 0.74	9.1 ± 0.64	10.14 ± 0.57	11.28 ± 0.44	12.84 ± 1.48
COS/ZnO ^d^	1 ± 0.28	3.33 ± 0.34	6.32 ± 0.27	8.71 ± 0.65	9.62 ± 0.47	11.19 ± 0.81	12.02 ± 0.38
CMCS/ZnO ^e^	0.81 ± 0.31	3.19 ± 0.26	6.14 ± 0.20	8.31 ± 0.52	9.25 ± 0.73	10.88 ± 0.39	12.15 ± 0.82

^a^ Numbers after ± represent the standard deviation. ^b^ K represents untreated dyed wood. ^c^ CS/ZnO represents chitosan and ZnO composite-treated dyed wood. ^d^ COS/ZnO represents chitooligosaccharide and ZnO composite-treated dyed wood. ^e^ CMCS/ZnO represents carboxymethyl chitosan and ZnO composite-treated dyed wood.

**Table 2:** Comparison of ∆E changes after irradiation for treated dyed wood with other woods.

Wood	Reduction of ∆E Compared to Untreated Materials (%)	Processing Method	Test Conditions	Reference
Dyers’s Oleander	55%	Propylene glycol, ZnO coating	60 °C, 340 nm, 0.68 W/m^2^, 500 h	[18]
European spruce	32.5%	Chitosan/CeO_2_ coating	45 °C, relative humidity of 50%, 420 nm, 1 W/m^2^, 400 h	[36]
Norway spruce	39.8%	Wax/ZnO	40 °C, 315–400 nm, 240 h	[37]
-	47.8%	ZnO/styrene-acrylic coating	60 °C, 313 nm, 0.71 W/m^2^, 480 h	[38]
Albizia lebbeck	54.2%	Thermal modification	60 °C, 340 nm, 0.68 W/m^2^, 500 h	[39]
Rubberwood	23.7%	Isopropenyl acetate modification	60 °C, 340 nm, 0.68 W/m^2^, 250 h	[40]
Dyed poplar wood	56.4%	Carboxymethyl chitosan/ZnO coating	50 °C, relative humidity of 55%, 280–1100 nm, 550 W/m^2^, 50 h	This work

**Table 3:** FTIR spectral band assignments of surface functional groups of dyed wood and treated wood before and after UV irradiation.

Wavenumber (cm^−1^)	Band Assignment
2957	C-H stretching in alkane
1726, 1734	C=O stretching of the non-conjugated carbonyl group
1504, 1590	C=C stretching vibration of the aromatic skeleton
1233	-OH stretching vibration in the benzene ring
1160	C-O stretching in the ester group

**Table 4:** Factors and levels of the assay design.

Factors	Levels		
Types of cationic coatings	Chitosan	Carboxymethyl chitosan	Chitosan oligosaccharides
ZnO concentration for anionic coatings	0.5%	1%	2%

## Data Availability

Not applicable.
